# Effects of Dietary Leucine Levels on Growth Performance and Muscle Quality in Oriental River Prawn (*Macrobrachium nipponense*)

**DOI:** 10.3390/biology15161333

**Published:** 2026-08-07

**Authors:** Shiqian Cao, Guodong Gao, Qianhui Wang, Bo Liu, Qunlan Zhou

**Affiliations:** 1 Wuxi Fisheries College, Nanjing Agricultural University, Wuxi 214128, China; 2024113023@stu.njau.edu.cn (S.C.); 18027446119@163.com (G.G.); liub@ffrc.cn (B.L.); 2 School of Fisheries and Life Science, Dalian Ocean University, Dalian 116023, China; 17690505665@163.com; 3 Key Laboratory of Aquatic Animal Nutrition and Health, Freshwater Fisheries Research Center, Chinese Academy of Fishery Sciences, Wuxi 214081, China

**Keywords:** *Macrobrachium nipponense*, leucine, growth performance, muscle quality, nutritional metabolism

## Abstract

This investigation assessed how varying dietary leucine concentrations impact the growth performance, antioxidant status, and muscle quality of the oriental river prawn (*Macrobrachium nipponense*). Six isonitrogenous and iso-lipidic diets containing incremental leucine levels (0.76%, 1.16%, 1.48%, 1.94%, 2.32%, 2.74%) were fed to prawns over 8 weeks. Dietary leucine significantly affected measured growth indices (*p* < 0.05). Based on the broken-line regression model of specific growth rate (SGR) and protein deposition rate (PDR), the optimal dietary leucine levels were 2.36% and 2.34%, corresponding to 6.45% and 6.39% of crude protein. Crude protein and muscle leucine contents peaked at the 2.32% leucine level, significantly exceeding those in the 1.16% group (*p* < 0.05). Hepatopancreatic lipase (LPS) activity and total antioxidant capacity (T-AOC) were significantly elevated in the 2.74% group relative to the control (*p* < 0.05). Dietary leucine levels significantly affected superoxide dismutase (SOD) and malondialdehyde (MDA) (*p* < 0.05); the maximum SOD activity was observed in the 1.94% group, while the minimum MDA level was observed in the 2.32% group. Drip loss was significantly reduced by dietary leucine supplementation (*p* < 0.05). Dietary leucine significantly affected gene expression related to protein and muscle synthesis in muscle (*p* < 0.05). Relative mRNA levels of *tor* and *s6k1* were significantly elevated in the 2.32% and 2.74% groups compared with the control (*p* < 0.05), while *myf5* expression peaked at 2.74% leucine (*p* < 0.05). Conversely, *mstn* expression was significantly downregulated at 2.32% leucine relative to the 1.16% group (*p* < 0.05). These results demonstrate that an appropriate dietary leucine level can promote prawn growth, enhance antioxidant defense capacity, and improve muscle quality.

## Simple Summary

This study investigated the impact of varying dietary leucine concentrations on the growth and muscle quality of the oriental river prawn. The primary objective was to find the optimal leucine level for prawn farming and to elucidate its impacts on muscle quality. Results showed that incorporating an appropriate leucine concentration (about 2.34–2.36% of the diet) significantly improved prawn growth, boosted antioxidant defenses, and reduced muscle drip loss. The study concluded that moderate leucine levels were correlated with the expression of genes involved in the target of rapamycin (TOR) and myogenesis. These findings assist farmers in producing healthier prawns with superior meat quality, thereby enhancing farm efficiency and product value and benefiting both the aquaculture industry and consumers.

## 1. Introduction

A balanced provision of essential amino acids in the diet is crucial for the intensive culture of the oriental river prawn (*Macrobrachium nipponense*), as it directly impacts their growth performance, metabolic efficiency, and product quality. Leucine, a key branched-chain amino acid for aquatic organisms, plays an essential role in modulating growth, metabolic processes, and muscle physicochemical properties [1,2]. It acts as a rate-limiting substrate for protein synthesis in living organisms and has been identified as a key regulator of this process. Accumulating evidence has shown that dietary leucine is associated with the target of rapamycin (TOR) gene expression, thereby influencing growth and protein synthesis, as has been validated in studies on largemouth bass (*Micropterus salmoides*) and Pacific white shrimp (*Litopenaeus vannamei*) [3,4]. In addition, empirical studies have established that dietary leucine fortification enhances growth indices, feed utilization efficiency, and immunological competence across multiple aquatic taxa. For example, dietary leucine supplementation at optimal concentrations has been documented to enhance growth metrics in the swimming crab (*Portunus trituberculatus*), manifesting as elevated weight gain and improved feed conversion efficiency [5]. Furthermore, leucine elevates whole-body protein content, promotes protein accretion in Pacific white shrimp and augments weight gain in tiger shrimp (*Penaeus monodon*) [4,6]. Currently, existing research has primarily focused on the arginine, threonine, and tryptophan requirements of the oriental river prawn [7,8,9], leaving the leucine requirement inadequately understood.

Leucine was shown to act as a pivotal signaling molecule that modulates the mammalian target of rapamycin (mTOR) pathway, a central axis coordinating protein synthesis and cell proliferation [10,11]. Through the TOR pathway, mRNA translation initiation was promoted by leucine, which consequently enhanced protein synthesis and reduced protein degradation within tissues [12]. Among branched-chain amino acids (BCAAs), leucine was identified as a key regulator of multiple metabolic processes. In Pacific white shrimp [13], dietary supplementation with leucine resulted in enhanced activities of trypsin and lipase, which are critical indicators of digestive enzyme activity; moreover, the antioxidant capacity was improved, as evidenced by an increase in superoxide dismutase (SOD) activity and a decrease in malondialdehyde (MDA) levels. Similarly, in triploid rainbow trout (*Oncorhynchus mykiss*) and juvenile yellowtail amberjack (*Trachinotus ovatus*), dietary leucine levels were found to regulate their redox state [14,15]. However, most of the available evidence was derived from fish, whereas relevant research remained scarce in commercially important decapod crustaceans, particularly freshwater prawn.

The impact of leucine on muscle quality has emerged as a focal point in nutritional research, given that muscle quality is a critical determinant of the commercial value of aquatic products. Muscle quality characteristics, such as water-holding capacity, textural properties, and amino acid composition, are closely linked to consumer acceptance and market pricing [16]. The myogenic regulatory factors (MRFs) represent a functionally significant transcription factor family that governs both the proliferation and terminal differentiation of muscle satellite cell populations. Key members of this family include myodulin (*myod*), myogenic factor 5 (*myf5*), muscle regulatory factor 6 (*myf6*), and myogenin (*myog*), all of which are integral to muscle development and maintenance [17]. Conversely, myostatin (*mstn*) exerts suppressive control over muscular hypertrophy by restraining the proliferative and differentiative capacities of crustacean myocytes [18,19]. Leucine has been shown to promote muscle development through enhancing myoblast proliferation and differentiation, an effect mediated through the regulation of myogenic factors including *myf5* and *mstn* [20,21]. Furthermore, because leucine serves as a biosynthetic precursor for glutamine and alanine, its availability directly impacts the accumulation of flavor-contributing amino acids in muscle tissue, consequently influencing both the sensory palatability and nutritional profile of aquatic food products. Research conducted on teleost species has confirmed that dietary leucine optimization elevates crude protein concentrations, increases muscle firmness, and improves water-holding capacity [22,23]. However, investigation into leucine’s regulation of crustacean muscle quality remains limited, and the molecular mechanisms linking dietary leucine levels to muscle physicochemical properties and flavor-related compounds require further exploration.

The oriental river prawn (*Macrobrachium nipponense*) possesses high nutritional value, exhibits rapid growth rates and strong environmental adaptability, and serves as an important aquaculture species in southeastern China. Considering leucine’s pivotal function in stimulating growth, modulating metabolic processes, and enhancing muscle quality, along with the limited comprehensive investigations regarding this amino acid in oriental river prawns, we designed and administered six distinct dietary treatments containing graded leucine concentrations to the test organisms. The aim was to identify the most suitable dietary leucine concentration for maximizing growth performance and feed conversion efficiency in this crustacean species. Furthermore, by measuring multiple antioxidant parameters, this study investigated the effect of adding leucine to the diet on the antioxidant stress resistance of the oriental river prawn. The investigation further evaluated how dietary leucine supplementation influenced the physicochemical properties, amino acid profiles, and flavor-determining parameters of prawn muscle tissue. These findings are expected to provide a scientific basis for the rational and effective application of leucine in aquacultural practices, with the aim of elevating both productivity and product quality in oriental river prawn farming.

## 2. Materials and Methods

### 2.1. Experimental Diets

Six isonitrogenous (with crude protein of 36.5%) and iso-lipidic (with crude lipid of 7.2%) semi-purified experimental diets were prepared, employing fish meal, soybean protein concentrate, and crystalline amino acid mixture as nitrogenous ingredients, while fish oil, soybean oil, and soybean lecithin served as lipid ingredients. As usual, non-essential glycine was used to replace graded-level leucine on an equal nitrogen basis to maintain isonitrogenous among different experimental diets. Leucine was added to these formulations at graded inclusion rates of 0.62%, 1.02%, 1.42%, 1.82%, 2.22%, and 2.62%, which corresponded to analyzed values of 0.76%, 1.16%, 1.48%, 1.94%, 2.32%, and 2.74%, respectively. All ingredients were pulverized to fine particles and then screened through an 80-mesh sieve. Vitamins, minerals, and amino acid premixes were blended thoroughly via sequential dilution. Thereafter, lipid components were introduced, after which water was slowly poured in (500 mL/kg) to obtain a homogeneous blend. The resulting mixtures were processed through a twin-screw extruder (F-26, manufactured by Guangzhou Huazhong Optical Mechanical and Electrical Technology Co., Ltd., Guangzhou, China) to produce 1.0 mm diameter pellets, and then they were dried under ambient conditions without direct sunlight exposure for 48 h, and finally preserved at −20 °C. The feed formulation is shown in Table 1.

### 2.2. Experimental Prawns and Rearing Management

Juvenile specimens of oriental river prawns were sourced from the Freshwater Fisheries Research Center (FFRC), Chinese Academy of Fishery Sciences (CAFS). A total of 1440 individuals displaying complete appendages, normal behavioral responses, vigorous feeding activity, and comparable body masses (initial mean weight 0.21 ± 0.01 g) were chosen for the trial. The entire experiment was conducted at the Dapu base of FFRC in Yixing. These prawns were allocated at random into six treatment groups, with every group containing four independent replicates (60 prawns per replicate), and subsequently maintained in 24 circular rearing units (180 cm in diameter and 100 cm in depth). Each rearing unit was equipped with artificial plastic leaves as shelter. Aeration was maintained continuously to sustain dissolved oxygen concentrations exceeding 5 mg/L. Water chemistry indicators were assessed at two-week intervals, maintaining total ammonia nitrogen below 0.2 mg/L, pH within 7.0–8.5, and thermal conditions between 25 and 28 °C. Before initiating the formal trial, the test organisms completed a seven-day adaptation phase receiving standard commercial feed. The formulated diets were offered three times per day (7:30, 12:30, and 17:00) at a ration equivalent to 3% of the prawns’ biomass. Uneaten feed and excreta were removed via siphoning one-hour post-feeding. The entire culture duration extended over eight weeks.

### 2.3. Sample Collection

Before harvesting specimens, the prawns were deprived of feed for 24 h. To evaluate growth performance, survival counts and biomass measurements were documented for each tank. Specimens selected for processing were first immobilized through cold-water anesthesia. From every tank, hepatopancreatic tissues were dissected from nine individuals and weighed for the hepatosomatic index (HSI) and condition factor (CF). Two pooled hepatopancreas samples were collected from 8 prawns per tank with 4 individuals pooling to one sample (that was 8 pooled samples per treatment) which were stored at −20 °C for determination of digestive enzyme and antioxidant enzyme activities.

For muscle quality assessment, the prawns were decapitated and deshelled. Four individual prawns per tank were pooled to be one sample, resulting in four pooled samples per treatment, which were subsequently stored at −20 °C for proximate composition analysis. An additional set of four individuals from the same tank were pooled to create another four pooled muscle samples per treatment for amino acid profile analysis. Furthermore, four individuals were pooled with two individuals in one pool, resulting in eight pooled samples per treatment, which were used for the determination of lactic acid (LA), lactate dehydrogenase (LDH), and glucose (GLU) levels. For texture profile analysis, four individuals from each tank were used, with two allocated for shear force measurement and the remaining two for other texture parameters. Additionally, four fresh individuals per tank were prepared for water-holding capacity assessment, with two designated for drip loss and two for cooking loss. Finally, two individual muscle samples per tank, totaling eight individual samples per treatment, were stored at −80 °C for muscle gene expression analysis.

### 2.4. Proximate Composition and Amino Acid Analysis

Diet and muscle proximate composition were determined following the AOAC guidelines [24]. Moisture was measured for 2 h until it reached a constant weight; crude protein was estimated using the Kjeldahl procedure; lipid fractions were recovered through the Soxhlet extraction; and mineral residues were quantified via combustion at 560 °C for a 5 h duration.

The amino acid content in feed and muscle was analyzed as per Muhammad et al. [25]. In short, 0.1 g of the sample was hydrolyzed with 6 mol/L HCl at 130 °C for 7 h and then diluted to 100 mL with distilled water. A 1 mL portion was freeze-dried, reconstituted in 0.02 mol/L HCL, and then filtered through a 0.22 µm membrane. The amino acids were then detected using a Hitachi L-8900 automatic analyzer (Tokyo, Japan). The amino acid composition of experimental feeds was shown in Table 2.

### 2.5. Digestive and Antioxidant Enzyme Activity Analysis

Approximately 0.1 g of hepatopancreas was homogenized with 9 mL 0.85% normal saline using a high-throughput tissue disruptor (SCIENTZ-48, Ningbo Scientz Biotechnology Co., Ltd., Ningbo, China). The protein concentration was determined using the Coomassie Blue method on the supernatant of the homogenate, and enzyme activities, including α-amylase (AMS), trypsin (TRY), lipase (LPS), SOD, MDA, and total antioxidant capacity (T-AOC), were determined using commercial assays (Nanjing Jiancheng Bioengineering Institute, Naning, China) following the manufacturer’s instructions.

### 2.6. Muscle Physicochemical Property Analysis

Lactic acid (LA), lactate dehydrogenase (LDH), and glucose (GLU) were assessed with commercial assay kits (Nanjing Jiancheng Bioengineering Institute, Naning, China). The muscle water-holding capacity was assessed by drip loss and cooking loss according to the method reported by He et al. [26]. Texture profile analysis (TPA) and shear force were measured using a Universal TA texture analyzer (TA. XT Plus C, Stable Micro System Co., Ltd., Godalming, UK). The test conditions for the TPA and shear force were determined using the method described in a previous study [27]. For shear force measurement, a blade (20 mm wide and 1 mm thick) was used at a test speed of 1 mm/s. For TPA, a cylindrical flat-bottomed probe (25 mm diameter) was employed at a test speed of 1 mm/s with a 5 s dwell time between compressions.

### 2.7. Gene Expression Analysis

Total RNA was extracted from muscle using RNAiso plus kits (Takara Co., Ltd., Dalian, China), and the RNA concentration was determined using a Nanodrop spectrophotometer (Thermo Scientific, Wilmington, DE, USA). cDNA was synthesized from 500 ng/uL RNA using the Prime Script™ RT Master Kit (Takara Co., Ltd., Dalian, China). All primers (Table 3) were synthesized by Generay Biotechnology Co., Ltd. (Shanghai, China). Real-time quantitative PCR was conducted using a real-time quantitative PCR instrument (Bio-Rad Laboratories, Inc, Hercules, CA, USA) with the TB Green II Fluorescent Reagent Kit (Takara Co., Ltd., Dalian, China). The reaction volume was 20 μL. *β-actin* was selected as the internal reference gene. Relative gene expression levels were quantified using the 2^−ΔΔCT^ method [28].

### 2.8. Statistical Analysis

All statistical computations were carried out in SPSS 20.0. After confirming normality and homogeneity of variances, one-way ANOVA was used to assess differences in growth performance, approximate muscle composition, and amino acid composition among treatments, with post hoc comparisons made using Tukey’s test (*p* < 0.05). The other data were analyzed using linear mixed-effects models, with treatment as a fixed effect and tank as a random effect to correct for the non-independence of subsamples within the same tank. Post hoc multiple comparisons were performed using Tukey’s test (*p* < 0.05). Data were reported as mean ± standard error (Mean ± S.E.M.). Figures were created using Origin 2024 and GraphPad Prism 8.0 software. Linear regression analysis was conducted based on the data of four tank replicates to establish the relationships between specific growth rate (SGR) and protein deposition rate (PDR) and dietary leucine levels. The “segmented” package in R (2025) was used to estimate standard errors and confidence intervals for breakpoints. Additionally, orthogonal polynomial contrasts were employed to examine the linear and quadratic responses of the measured indices to graded dietary leucine levels. The coefficient of determination (R^2^) was adopted to evaluate the goodness of fit of each regression model, and the model with the highest R^2^ was selected to determine the optimal leucine requirement.

## 3. Results

### 3.1. Growth Performance and Feed Utilization

The growth parameters in oriental river prawns are presented in Table 4. Significant variations among treatments were detected for final weight (FW), SGR, weight gain rate (WGR), feed conversion ratio (FCR), protein efficiency ratio (PER), PDR, and HSI (*p* < 0.05). Conversely, survival ratio (SR) and CF exhibited no statistically significant divergence across all experimental units (*p* > 0.05). FW, SGR, and WGR likewise attained their maxima at the 2.32% leucine concentration and were significantly superior to those recorded for the 0.76% and 1.16% leucine treatments (*p* < 0.05). With respect to feed utilization, the minimal FCR was recorded in the 2.32% leucine treatment, which was significantly lower than that of the 0.76%, 1.16% and 2.74% leucine treatments (*p* < 0.05). The PER was highest in the 2.32% group and significantly higher than in all other groups except the 1.94% group (*p* < 0.05). The highest PDR was achieved in the 2.32% leucine treatment, significantly higher than all remaining treatments (*p* < 0.05). Furthermore, HSI reached its maximum in the 2.32% leucine treatment, displaying a significant increase relative to the basal and 1.16% leucine treatments (*p* < 0.001).

**Figure 1:**
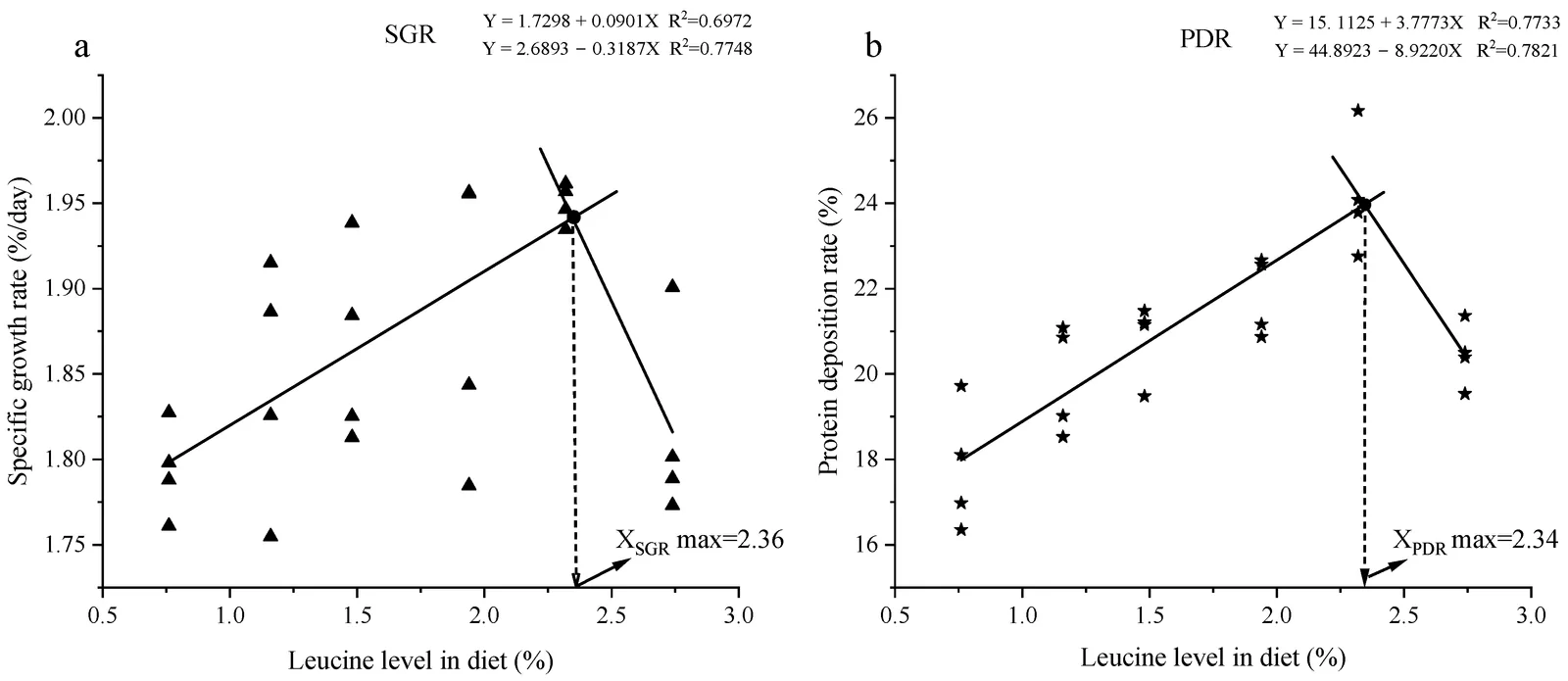
Broken-line regression analysis between dietary leucine levels and specific growth rate (**a**) protein deposition rate (**b**) of *M. nipponense.* Note: ▲, data of specific growth rate per tank, ★, data of protein deposition rate per tank. Data from each tank were plotted, with four replicates per treatment group.

Polynomial contrast analysis demonstrated that graded leucine inclusion produced pronounced quadratic responses in FW, SGR, WGR, FCR, PDR, and HSI (*p* < 0.05), whereas SR showed no statistically meaningful linear trends and CF showed no statistically meaningful cubic trends (*p* > 0.05). Linear broken-line regression and quadratic regression models were used to estimate the optimal dietary leucine level for the oriental river prawn. The broken-line regression model yielded the highest coefficient of determination R^2^; therefore, the broken-line regression model was selected to estimate the optimal leucine levels based on SGR and PDR. Using the linear regression model (Figure 1), two linear equations were derived for SGR: Y = 1.7298 + 0.0901X (R^2^ = 0.4862) and Y = 2.6893 − 0.3187X (R^2^ = 0.7748), with an optimal leucine content of 2.36% (crude protein content of 6.45%), SEM = 0.121, and 95% CI = [2.11, 2.61]; for PDR, the equations were Y = 15.1125 + 3.7773X (R^2^ = 0.7733) and Y = 44.8923 − 8.9220X (R^2^ = 0.7821), with an optimal leucine content of 2.34% (crude protein content of 6.39%), SEM = 0.078, 95%, and CI = [2.18, 2.50] (Figure 1).

### 3.2. Digestive Enzyme and Antioxidant Enzyme Activities

**Figure 2:**
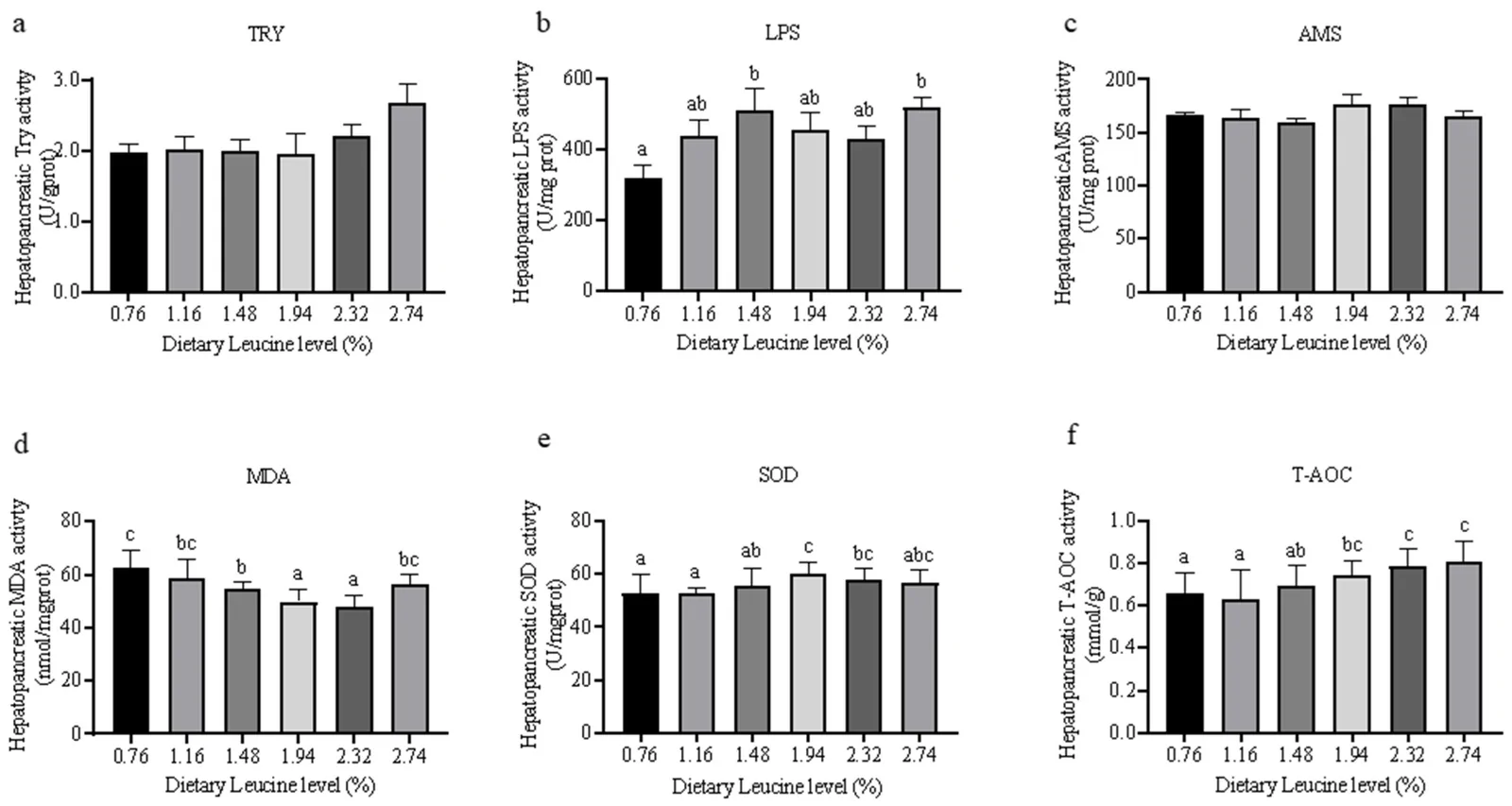
The effect of dietary leucine levels on the activity of digestive and antioxidant enzymes in hepatopancreas of *M. nipponense*. Note: (**a**), TRY = trypsin; (**b**), LPS = lipase; (**c**), AMS = amylase; (**d**), MDA = malondialdehyde; (**e**), SOD = superoxide dismutase; (**f**), T-AOC = total antioxidant capacity. Values are means ± S.E.M (n = 4). The different top letters of the bar chart indicate significant differences (*p* < 0.05), while the unmarked letters indicate no significant differences (*p* > 0.05).

Graded leucine supplementation exerted no statistically meaningful impact on AMS and TRY enzymatic activities in prawn hepatopancreatic tissues (*p* > 0.05) (Figure 2). Nevertheless, LPS activity was markedly modulated by leucine, with specimens receiving 1.48% and 2.74% leucine displaying substantially elevated LPS activity relative to the basal diet group (*p* < 0.05). Additionally, hepatopancreatic antioxidant parameters (SOD, MDA, T-AOC) were significantly altered by varying leucine concentrations (*p* < 0.05) (Figure 2). SOD activity attained its peak at the 1.94% leucine concentration, markedly surpassing levels detected in the 0.76% and 1.16% treatments (*p* < 0.05). For MDA concentrations, both the 1.94% and 2.32% leucine treatments demonstrated marked reductions compared to the basal diet and all remaining leucine treatments (*p* < 0.05). Collectively, hepatopancreatic T-AOC activity demonstrated an upward trajectory with escalating leucine doses, reaching its apex in the 2.32% treatment, which exceeded that of the 0.76% treatment (*p* < 0.05).

### 3.3. Muscle Proximate Composition and Amino Acid Profile

The muscular proximate composition for oriental river prawns was summarized in Table 5. Moisture, crude protein, and ash showed no statistically significant divergence among treatments (*p* > 0.05). However, crude protein exhibited a positive dose–response relationship with dietary leucine, reaching its maximum in the 2.32% treatment. Likewise, crude lipid peaked in the 2.32% leucine treatment, significantly exceeding the 1.16% treatment (*p* < 0.05).

The amino acid composition of muscle was presented in Table 6. Total EAA content (ΣEAA) varied between 7.52% and 8.29%, with the peak concentration documented in the 2.32% leucine treatment. Muscular leucine concentrations demonstrated a positive correlation with dietary leucine inclusion rates. Notably, muscular leucine level from the 2.32% group was exhibiting significantly higher leucine levels than those from the 0.76%, 1.16%, 1.48%, and 1.94% groups (*p* < 0.05), with the exception of the 2.74% group. The ΣEAA/ΣNEAA ratio fluctuated between 0.87 and 0.93, with no statistically meaningful variation detected across the experimental units (*p* > 0.05).

### 3.4. Muscle Physicochemical Properties

**Figure 3:**
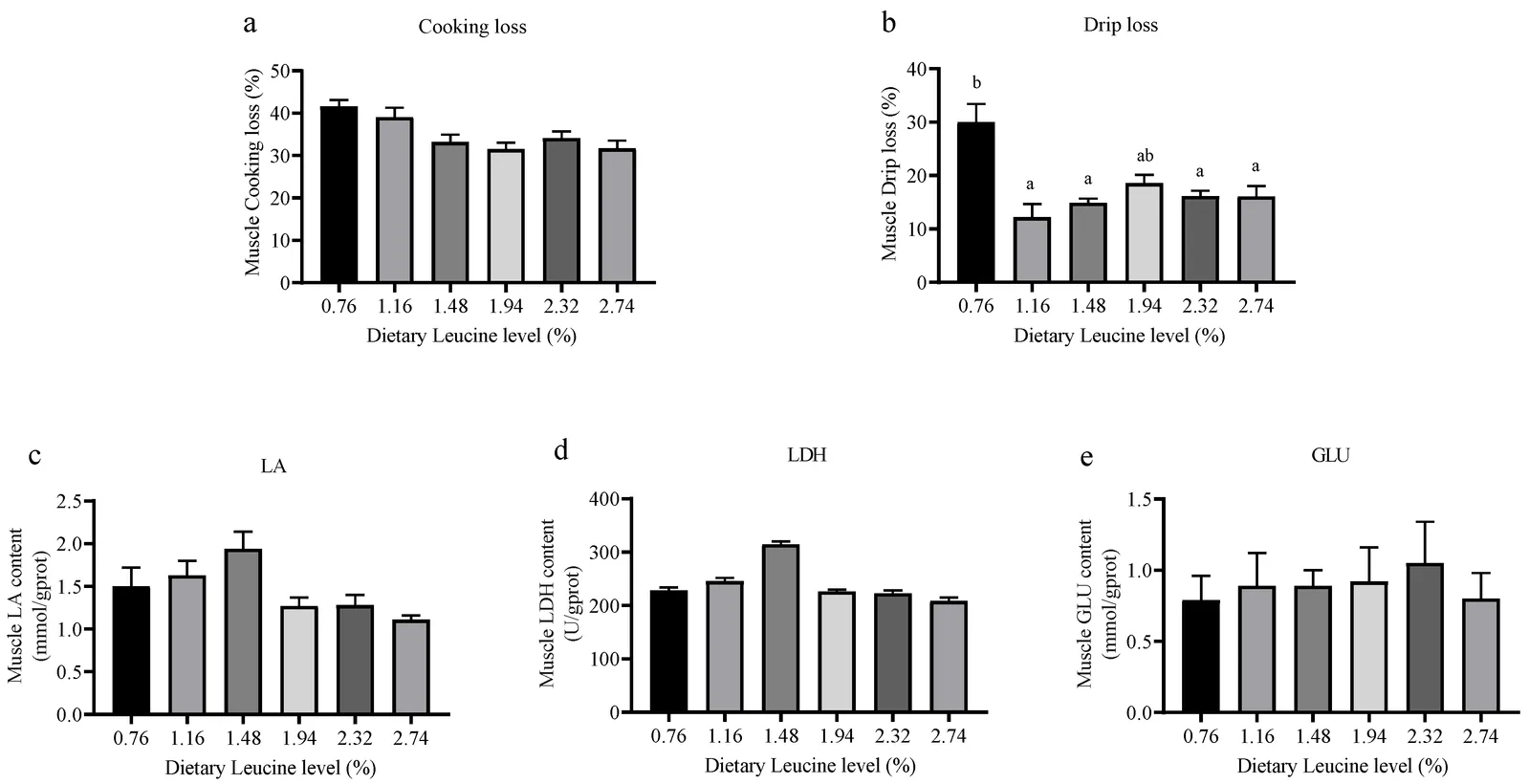
The effect of dietary leucine levels on water-holding capacity and the muscle chemical composition of *M. nipponense*. Note: (**a**), Cooking loss (%) = 100 × (Pre-cooking weight − post-cooking weight)/(Pre-cooking weight); (**b**), Drip loss (%) = 100 × (Initial sample mass − post-storage mass)/(Initial sample mass); (**c**), LA = lactic acid; (**d**), LDH = lactic dehydrogenase; (**e**), GLU = glucose. Values are means ± S.E.M (n = 4). The different top letters of the bar chart indicate significant differences (*p* < 0.05), while the unmarked letters indicate no significant differences (*p* > 0.05).

The water-holding capacity of muscle, together with LA, LDH, and GLU concentrations in muscular tissue, are depicted in Figure 3. Dietary leucine concentrations exerted a statistically significant influence on the drip loss of the oriental river prawn musculature (*p* < 0.05), while they had no significant effect on other parameters (*p* > 0.05). The drip loss was markedly reduced in the remaining leucine treatments compared to the 0.76% treatment (*p* < 0.05).

The effects of leucine on the muscle texture were shown in Table 7. Graded leucine supplementation exerted statistically meaningful effects on muscular cohesiveness, elasticity, and shear resistance in oriental river prawns (*p* < 0.05). Nevertheless, hardness, springiness, gumminess, and chewiness remained unaffected by these dietary manipulations (*p* > 0.05). The 1.94% treatment exhibited the lowest adhesiveness, substantially below that of the 1.48% treatment (*p* < 0.05), while simultaneously presenting the maximal elasticity, significantly exceeding the basal diet group (*p* < 0.05). Shear force measurements displayed a downward trend with escalating leucine doses, with minima documented in both the 2.32% and 2.74% treatments, both of which were markedly reduced relative to the 0.76% treatment (*p* < 0.05).

### 3.5. Expression of Protein Biosynthesis and Myogenic Regulatory Factors

**Figure 4:**
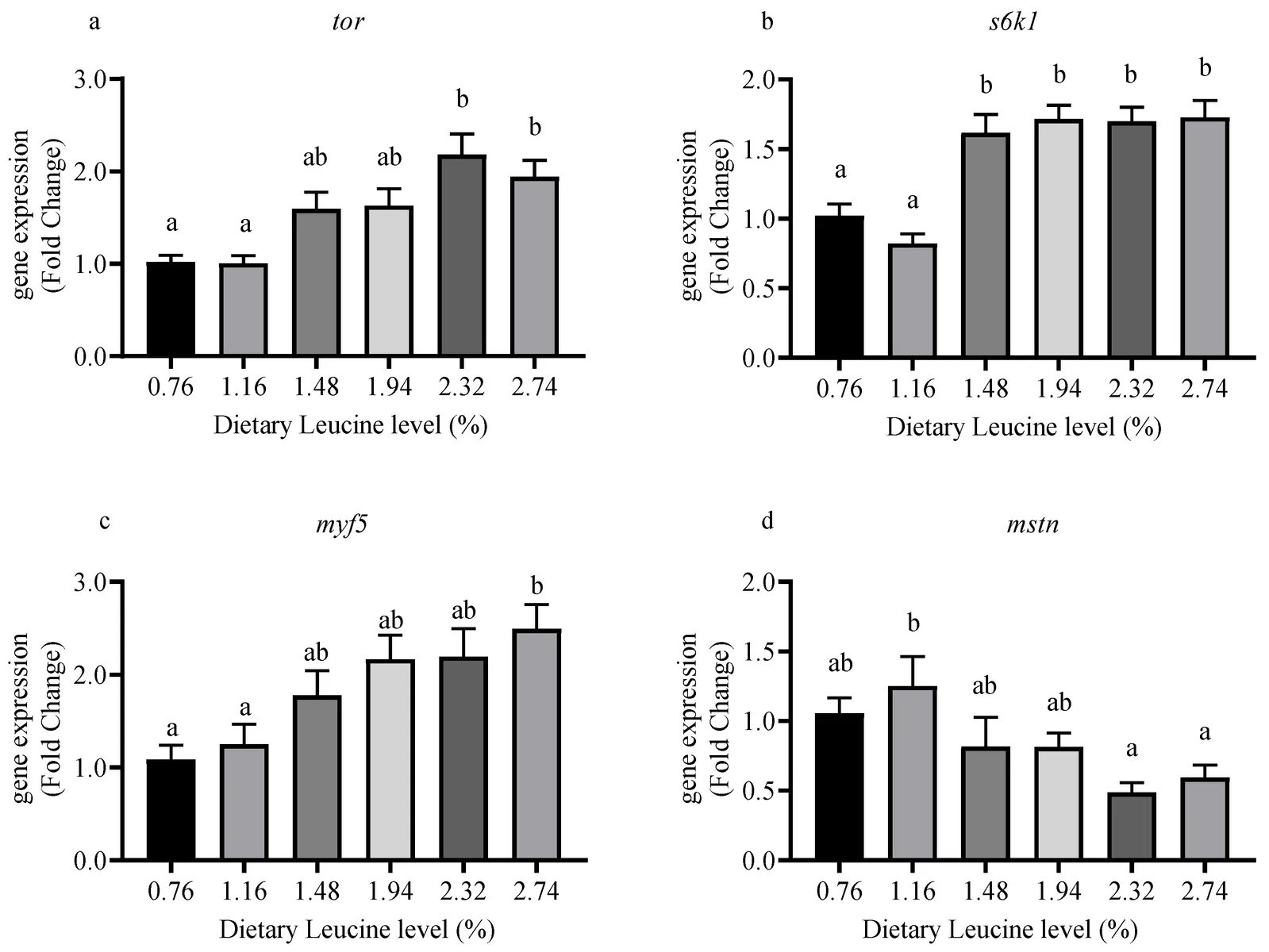
The effect of dietary leucine levels on expression of metabolic and myogenic regulatory factor genes in the muscle of *M. nipponense*. Note: Values are means ± S.E.M (n = 4). (**a**), *tor* = target of rapamycin; (**b**), *s6k1* = ribosomal protein S6 kinase beta-1; (**c**), *myf5* = myogenic factor 5; (**d**), *mstn* = myostatin. The different top letters of the bar chart indicate significant differences (*p* < 0.05), while the unmarked corner letters indicate no significant differences (*p* > 0.05).

Dietary leucine concentrations also exerted statistical effects on muscular protein biosynthesis and myogenic regulation in oriental river prawns (Figure 4). Specifically, *tor* and *s6k1* relative expressions were markedly elevated in the 2.32% and 2.74% treatments compared to both the basal diet and 1.16% treatment (*p* < 0.05). Concurrently, *myf5* mRNA levels exhibited a progressive rise with increasing leucine inclusion, attaining its maximum in the 2.74% treatment and markedly exceeding levels in the basal diet and 1.16% treatment (*p* < 0.05). Conversely, *mstn* relative expression followed an opposing trajectory, progressively declining as leucine supplementation intensified. The *mstn* mRNA levels for this negative regulator were markedly reduced in the 2.32% and 2.74% treatments relative to both the basal diet and 1.16% treatment (*p* < 0.05).

## 4. Discussion

### 4.1. Optimal Dietary Leucine Requirement Analysis

The present study demonstrated that dietary leucine supplementation significantly increased WGR and SGR values in oriental river prawns, which was consistent with findings reported for Pacific white shrimp [32]. Furthermore, appropriate leucine supplementation effectively reduced FCR and significantly elevated PDR values, a finding that parallels observations reported for Chinese mitten crabs (*Eriocheir sinensis*) [33]. However, some studies have indicated that leucine restriction significantly affects survival rate; for example, a similar trend was observed in fruit flies (*Drosophila melanogaster*), in which leucine restriction reduced viability [34]. In the present study, no significant differences in survival rate were detected among treatments.

Broken-line modeling of growth performance metrics estimated the most favorable dietary leucine concentrations for maximizing SGR and PDR at 2.36% and 2.34% of the formulation, respectively (corresponding to 6.45% and 6.39% of dietary protein). This range of values is consistent with findings reported for other commercially important decapod crustaceans. For Pacific white shrimp the dietary leucine requirement has been estimated at 2.36% to 2.48% of the diet [32]; for swimming crabs an optimal level of 2.27% has been reported [5]; and for Chinese mitten crabs, the optimal leucine level was determined to be 2.36% [35]. These comparisons suggest that leucine requirements among decapod crustaceans generally range from approximately 2.2% to 2.5% of the diet. In contrast, fish species indicated slightly higher leucine requirements. Juvenile grouper (*Epinephelus coioides*) required 3.51–4.45% leucine [36], golden pompano required 2.93–3.29% [37], and triploid rainbow trout required 3.03–3.05% [38]. Such interspecific divergence in leucine requirements presumably reflects phylogenetic distinctions, given that fish represent a more evolutionarily advanced lineage relative to crustaceans.

It should be noted that glycine, the non-essential amino acid used to replace leucine, decreased substantially across diets (from 4.66% to 2.44%), and cystine also showed a slight decline. However, since glycine and cystine were non-essential and could be synthesized endogenously, their reduction was not expected to constrain protein synthesis or growth. In the study of swimming crabs and leucine, glycine was also used as a substitute amino acid [5]. Furthermore, methionine can be endogenously converted into cystine via the trans-sulfuration pathway. In the present study, dietary methionine concentrations ranged from 0.96% to 1.03%, substantially exceeding the dietary methionine requirement of Chinese mitten crabs (0.70% of the diet) [39]. Accordingly, the supply of sulfur amino acids remained sufficient even in the treatment with the lowest cystine concentration. All other essential amino acids, especially isoleucine and valine, were maintained at consistent levels across treatments, with the exception of leucine. Although the experimental diets were formulated to be iso-lipidic, analyzed crude lipid concentrations varied from 7.66% to 8.98%. The diet containing 2.32% leucine exhibited the highest lipid content and achieved the optimal growth performance, and such variation may represent a potential confounding factor.

### 4.2. Effects of Leucine on Hepatopancreatic Enzyme Activities

The hepatopancreas serves as the primary metabolic organ in crustaceans, playing a crucial role in nutrient digestion, absorption, and antioxidant defense mechanisms. The present investigation demonstrated that dietary leucine fortification significantly affected both digestive and antioxidant enzymatic activities within the hepatopancreatic tissues of oriental river prawns. Notably, the activities of LPS were significantly elevated in the groups receiving 2.74% leucine compared to the control group, indicating that leucine promotes lipid digestion and absorption. These findings align with previous research on oval pomfret (*Trachinotus ovatus*), which demonstrated that dietary leucine enhances digestive enzyme activities and improves nutrient utilization [37]. Conversely, no significant effects of leucine were observed in AMS and TRY activities, suggesting that leucine may exert a more targeted regulatory effect on lipid digestion rather than on carbohydrate or protein digestion in oriental river prawns.

With respect to antioxidant parameters, maximal SOD activity was documented in specimens receiving 1.94% leucine, whereas MDA concentrations were significantly diminished in the 2.32% leucine treatments. These outcomes suggest that judicious leucine concentrations bolster the oxidative resistance of oriental river prawns by stimulating enzymatic antioxidants and suppressing lipid peroxidation. Comparable results have been documented for grass carp (*Ctenopharyngodon idella*) and rainbow trout [40,41]. Moreover, the significant increase in T-AOC in the 2.74% treatment further supports the hypothesis that optimal leucine fortification safeguards hepatopancreatic integrity by ameliorating oxidative insult and cellular injury [42,43].

### 4.3. Effect of Leucine on the Muscle Composition and Texture of the Oriental River Prawns

Muscle amino acid content is widely acknowledged as a key indicator for assessing muscle nutritional value [44]. The present study showed that muscular leucine content was influenced by dietary leucine levels, with the highest value observed at 2.32% leucine. However, no further increase was detected at 2.74% leucine, and there was no significant linear correlation between muscle leucine content and dietary leucine level across the entire dose range. This pattern is consistent with findings reported for triploid rainbow trout [14], Chinese mitten crabs [33] and blunt snout bream [20]. Nevertheless, although leucine could influence muscle leucine content, it did not act in a simple dose-dependent manner. This phenomenon may be attributable to interconversion between amino acids or to feedback regulation of leucine metabolism at higher dietary levels.

The present investigation revealed that graded leucine fortification significantly improved the muscular texture of oriental river prawns. Specifically, in terms of water-holding capacity, leucine supplementation significantly reduced drip loss in most treatment groups, with the lowest value observed at 1.16% leucine. Comparable outcomes have been reported in largemouth bass [21]. Regarding muscle texture, the 2.32% leucine group displayed the lowest muscle hardness and shear force, whereas the 1.94% leucine group achieved the highest springiness and lowest cohesiveness. These alterations in texture parameters suggest that leucine may improve the texture and water-holding capacity of oriental river prawn muscle, as inferred from physicochemical and texture indicators. These findings are consistent with observations reported in *Carassius auratus gibelio* and *Cyprinus carpio haematopterus* [45,46]. The observed improvements in muscle texture and water-holding capacity were inferred from physicochemical and texture indicators. Previous studies on other aquatic species have hypothesized that such changes may be related to alterations in muscle fiber density, fiber diameter, or connective tissue composition [21,47]. However, because none of these structural parameters were directly measured in the present study, these potential mechanisms remain speculative for oriental river prawns. Future investigations incorporating histological analysis and biochemical measurements are needed to determine whether these hypothesized mechanisms underlie the texture improvements observed in this study.

In terms of amino acid deposition, dietary leucine significantly increased muscle leucine content but exerted no effects on other essential amino acids or total amino acids. This indicates that muscle leucine concentration responds to dietary leucine supply under the present experimental conditions. Similar patterns have been observed in Pacific white shrimp [32] and grass carp [47], wherein dietary leucine addition was associated with enhanced amino acid retention in muscle and improved physicochemical properties of meat quality. The lack of pronounced effects on remaining amino acids presumably reflects the well-balanced amino acid formulation of the experimental diets, which likely averted competitive interactions between leucine and other branched-chain amino acids [48,49].

### 4.4. Effects on Protein Biosynthesis and Myogenic Regulatory Factors

The TOR signaling cascade is widely regarded as a pivotal modulator of cellular proliferation and protein biosynthesis across eukaryotic organisms. The present investigation demonstrated that graded leucine supplementation significantly induced *tor* and *s6k1* transcript abundance in the muscular tissues of oriental river prawns. These outcomes suggest that leucine is associated with the mRNA levels of the *tor* and *s6k1* genes, consequently related with protein biosynthesis and deposition. Concordantly, PDR values were significantly elevated in the 2.32% treatment relative to the basal diet. Parallel findings have been reported for Pacific white shrimp and juvenile golden pompano [32,37], where leucine was reported to be associated with TOR pathway stimulation and augmented muscular protein biosynthesis.

Myogenic regulatory factors (MRFS) and myostatin (MSTN) serve as pivotal modulators governing muscular development and hypertrophy. The present investigation demonstrated that graded leucine fortification significantly elevated *myf5* transcript abundance while concurrently suppressing *mstn* expression in the musculature of oriental river prawns. MYF5 is a key MRF known to be involved in myoblast proliferation and differentiation, whereas MSTN acts as a negative regulator of muscle growth [19]. In the present study, dietary leucine was associated with upregulation of *myf5* and downregulation of *mstn* transcript levels, suggesting a molecular environment permissive for muscle development; studies on largemouth bass have also yielded similar results [21]. However, because only mRNA abundances were measured, direct evidence for enhanced myoblast proliferation or inhibited muscle growth suppression should be investigated in further studies. The leucine-associated changes in *tor* and *s6k1* expression may contribute to the modulation of myogenic factors, given that prior investigations have established that TOR signaling can govern the expression of both MRFs and MSTN [2,23].

### 4.5. Feasibility and Limitations

From a practical perspective, the estimated leucine requirement of 2.34–2.36% of the diet is readily achievable in commercial feed formulation. Many conventional protein ingredients—including fish meal, soybean meal, and other plant protein sources—contain adequate levels of leucine to meet this requirement through careful ingredient balancing. For example, there have already been corresponding studies on both Pacific white shrimp and Chinese mitten crabs [4,34]. Crystalline leucine may also be added, when necessary, provided that appropriate feed processing techniques are employed to minimize dissolution losses [42]. However, the present study still has several limitations. The 8-week feeding trial conducted with juvenile prawns only detected selected gene markers; analyses of protein phosphorylation and tissue histology were not included. Furthermore, the estimated nutrient requirement parameters may be affected by feed formulations and culture conditions. Additional parameters related to muscle quality, such as histological analysis, collagen measurement, fiber diameter assessment, and sensory evaluation, should be considered in future studies to further elucidate the mechanisms by which leucine may enhance muscle quality. Accordingly, further verification under diverse experimental conditions is warranted.

## 5. Conclusions

In conclusion, dietary leucine levels were significantly associated with growth performance, metabolic homeostasis, and physicochemical properties of muscle in oriental river prawns. The optimal dietary leucine requirements for growth were estimated at 2.34–2.36% of the diet (6.39–6.45% of crude protein). These beneficial effects were accompanied by increased hepatopancreatic lipase activity and enhanced antioxidant enzyme activities, including elevated SOD and T-AOC and reduced MDA levels. Dietary leucine was also associated with changes in the expression of genes related to protein synthesis and myogenic regulation. Further functional studies, including protein phosphorylation and histological analyses, are warranted to elucidate the underlying mechanisms.

## Figures and Tables

**Table 1:** Feed formulation and proximate composition of the experimental feeds.

Ingredient	Dietary Leucine Levels (%)					
	0.76	1.16	1.48	1.94	2.32	2.74
Fish meal	10.00	10.00	10.00	10.00	10.00	10.00
Soybean protein concentrate	2.00	2.00	2.00	2.00	2.00	2.00
Squid paste	2.00	2.00	2.00	2.00	2.00	2.00
Amino acid mix ^a^	25.62	25.62	25.62	25.62	25.62	25.62
Oil ^b^	4.00	4.00	4.00	4.00	4.00	4.00
α-Starch	24.93	24.93	24.93	24.93	24.93	24.93
Carboxymethyl cellulose	6.00	6.00	6.00	6.00	6.00	6.00
Microcrystalline cellulose	15.00	15.00	15.00	15.00	15.00	15.00
Premix ^c^	2.00	2.00	2.00	2.00	2.00	2.00
Others ^d^	6.45	6.45	6.45	6.45	6.45	6.45
Glycine	2.00	1.60	1.20	0.80	0.40	0.00
Leucine	0.00	0.40	0.80	1.20	1.60	2.00
Total	100.00	100.00	100.00	100.00	100.00	100.00
Proximate composition of experimental diet (% dry matter)						
Moisture	7.39	6.73	6.76	6.72	7.31	7.00
Crude protein	37.07	36.61	36.92	36.77	35.81	36.51
Crude lipid	8.84	7.88	7.66	7.78	8.98	8.18
Ash	6.69	6.56	6.23	6.68	6.61	6.53
Leucine (% of dry diet)	0.76	1.16	1.48	1.94	2.32	2.74
Leucine (% of crude protein)	2.08	3.17	4.04	5.30	6.34	7.49

Note: ^a^ Amino acid mixture (225.62 g): glutamate 4.371 g, aspartic acid 3.153 g, lysine 2.392 g, arginine 2.387 g, alanine 1.944 g, glycine 1.920 g, isoleucine 1.477 g, valine 1.394 g, phenylalanine 1.222 g, tyrosine 1.011 g, threonine 0.926 g, proline 0.841 g, methionine 0.827 g, serine 0.618 g, histidine 0.527 g, cysteine 0.333 g, tryptophan 0.273 g, all of which were bought from Feer Co., Ltd. (Shanghai, China). ^b^ Oil was a mixture of fish oil and soybean oil with volume 1:1. ^c^ Premix (IU, g or mg kg^−1^ of diet): retinol 1.3 g, thiamine 3.2 g, riboflavin 2.1 g, pyridoxine 2.3 g, cobalamin 0.9 g, ascorbic acid 40.0 g, cholecalciferol 0.4 g, menaquinone 3.4 g, niacinamide 3.5 g, folate 0.3 g, D-biotin 0.7 g, inositol 12.0 g, boiling stone powder 350.5 g, sodium chloride 2.5 g, copper sulfate pentahydrate 12 g, magnesium sulfate heptahydrate 145 g, ferrous sulfate heptahydrate 64.8 g, zinc sulfate heptahydrate 33.7 g, potassium iodide 0.005 g, potassium chloride 22.3 g, sodium selenite 4.7 g, cobalt sulfate heptahydrate 1.5 g. ^d^ Others included monocalcium phosphate, 3%; soy lecithin, 2%; Vitamin C, 0.5%; choline chloride, 0.25%; DMPT, 0.5%; Ecdysone, 0.7%.

**Table 2:** Amino acid composition of experimental diets (g/100g of dry matter).

Amino Acid	Dietary Leucine Levels (%)					
	0.76	1.16	1.48	1.94	2.32	2.74
Essential amino acid						
Arginine	2.95	2.91	2.76	2.87	2.82	2.87
Histidine	0.66	0.68	0.68	0.66	0.65	0.64
Isoleucine	1.85	1.87	1.78	1.88	1.86	1.87
Leucine	0.76	1.16	1.48	1.94	2.32	2.74
Lysine	2.98	2.95	2.84	2.96	2.85	2.94
Methionine	1.03	1.02	0.97	1.01	0.96	1.01
Phenylalanine	1.67	1.65	1.59	1.66	1.64	1.63
Threonine	1.34	1.38	1.28	1.34	1.33	1.31
Valine	1.95	1.98	1.87	1.98	1.94	1.97
Non-essential amino acid						
Aspartic acid	4.33	4.34	4.16	4.34	4.33	4.29
Serine	0.95	0.91	0.89	0.93	0.90	0.91
Glycine	4.66	4.06	3.49	3.29	2.83	2.44
Alanine	2.60	2.57	2.46	2.58	2.54	2.56
Cystine	0.66	0.66	0.58	0.56	0.47	0.38
Tyrosine	0.80	0.85	0.80	0.80	0.77	0.80
Glutamic acid	6.26	6.28	5.98	6.27	6.19	6.20
Proline	1.31	1.21	1.12	1.22	1.26	1.25
Total	36.76	36.46	34.70	36.29	35.66	35.68

**Table 3:** RT-PCR primer sequences for genes on oriental river prawn.

Target Gene	Sequence (5′-3′)	Efficiency (%)	Accession Number /Reference
*tor*	F: AACAAGTCTCGTCCGTGTCC	96.27	XM064235143.1/[29]
	R: TTGAGCAGCTTCACGGCTTA		
*s* *6k1*	F: GGTCTTTGAGGGCTTTACG	112.04	XM064249887.1/[30]
	R: GTCCAGCAGAGTTTGGTGTAT		
*myf5*	F: AGACCTGGCAAGGAAAC	91.00	[31]
	R: GGAAGGCATCTGGAAAT		
*mstn*	F: CCACCCGATATGACAGG	122.36	[31]
	R: TGAGCGTGAAGCGTAGAT		
*β-actin*	F: GTGCCCATCTACGAGGGTTA	100.44	FL589653.1/[29]
	R: CGTCAGGGAGCTCGTAAGAC		

Note: *tor*, target of rapamycin; *s6k1*, ribosomal protein S6 kinase beta-1; *myf5*, myogenic factor 5; *mstn*, myostatin.

**Table 4:** Effect of leucine on growth performance and feed utilization of oriental river prawn ^1^.

Dietary Leucine Levels (%)		SR (%) ^2^	FW (g) ^3^	SGR (%/d) ^4^	WGR (%) ^5^	FCR ^6^	PER ^7^	PDR (%) ^8^	HSI (%) ^9^	CF ^10^
0.76		63.75 ± 2.19	21.33 ± 0.80 ^a^	1.80 ± 0.01 ^a^	173.06 ± 2.10 ^a^	2.43 ± 0.10 ^c^	1.01 ± 0.04 ^a^	17.79 ± 0.74 ^a^	9.96 ± 0.56 ^a^	1.59 ± 0.03
1.16		62.92 ± 1.85	21.18 ± 0.70 ^a^	1.85 ± 0.04 ^a^	181.26 ± 5.56 ^a^	2.19 ± 0.07 ^bc^	1.13 ± 0.04 ^ab^	19.87 ± 0.64 ^ab^	10.86 ± 0.43 ^ab^	1.61 ± 0.03
1.48		64.58 ± 2.75	22.60 ± 0.82 ^ab^	1.87 ± 0.03 ^ab^	184.32 ± 4.63 ^a^	2.06 ± 0.05 ^ab^	1.19 ± 0.03 ^b^	20.83 ± 0.46 ^b^	12.32 ± 0.70 ^abc^	1.61 ± 0.02
1.94		66.25 ± 3.15	23.73 ± 1.00 ^ab^	1.89 ± 0.05 ^ab^	187.62 ± 6.83 ^ab^	1.97 ± 0.04 ^ab^	1.24 ± 0.03 ^bc^	21.82 ± 0.47 ^b^	11.69 ± 0.52 ^abc^	1.66 ± 0.02
2.32		74.17 ± 2.50	26.98 ± 1.06 ^b^	1.95 ± 0.01 ^b^	198.01 ± 1.00 ^b^	1.83 ± 0.05 ^a^	1.38 ± 0.04 ^c^	24.20 ± 0.72 ^c^	13.44 ± 0.60 ^c^	1.65 ± 0.03
2.74		68.33 ± 4.14	23.78 ± 1.71 ^ab^	1.82 ± 0.03 ^ab^	176.59 ± 4.53 ^a^	2.12 ± 0.04 ^b^	1.16 ± 0.02 ^b^	20.45 ± 0.37 ^b^	12.91 ± 0.73 ^bc^	1.67 ± 0.03
Polynomial Contrasts	L ^11^	*	**	ns	ns	**	**	**	***	*
	Q ^12^	ns	*	ns	ns	**	**	**	***	*
	C ^13^	*	**	**	**	***	***	***	***	ns

Note: ^1^, Values are means ± SEM (n = 4). Means in the same column sharing different superscript letter are significantly different, as determined by Tukey’s test (*p* < 0.05). ^2^, SR: Survival = 100 × (final number of prawn/initial number of prawn). ^3^, FW: Final weight. ^4^, SGR: Specific growth rate (%/d) = 100 × (ln final wet weight (g) − ln initial wet weight (g))/duration (days). ^5^, WGR: Weight gain rate (%) = total wet weight gain/initial prawn total weight. ^6^, FCR: Feed conversion ratio = dry diet fed/wet weight gain. ^7^, PER: Protein efficiency ratio = wet weight gain/dry protein intake. ^8^, PDR: Protein deposition rate (%) = (final protein content − initial protein content)/protein intake. ^9^, HSI: Hepatopancreas somatic indices =100 × (hepatopancreas weight/body weight). ^10^, CF: Condition factor =100 × (final body weight of prawn/body length^3^). ^11^, L: linear trend. ^12^, Q: quadratic trend, ^13^, C: cubic. ns: not significant. The letters a, b and c indicate differences between treatments (*p* < 0.05); unmarked letters indicate no significant differences (*p* > 0.05). *: *p* < 0.05. **: *p* < 0.01. ***: *p* < 0.001.

**Table 5:** Effects of dietary leucine levels on muscle composition of oriental river prawn (% of w.w.).

Item	Dietary Leucine Levels (%)					
	0.76	1.16	1.48	1.94	2.32	2.74
Moisture	74.18 ± 0.06	73.32 ± 0.24	73.03 ± 1.17	74.25 ± 0.56	72.91 ± 0.72	74.03 ± 0.41
Crude protein	17.33 ± 0.30	18.17 ± 0.27	18.73 ± 0.91	18.91 ± 0.29	19.22 ± 0.22	18.54 ± 0.25
Crude lipid	3.94 ± 0.02 ^ab^	3.89 ± 0.06 ^a^	4.05 ± 0.13 ^ab^	4.20 ± 0.07 ^ab^	4.42 ± 0.22 ^b^	4.18 ± 0.06 ^ab^
Ash	3.01 ± 0.05	3.08 ± 0.07	3.08 ± 0.07	2.96 ± 0.08	3.15 ± 0.06	2.93 ± 0.05

Note: Values are means ± SEM (n = 4). Means in the same line with different superscripts are significantly different by Tukey’s test (*p* < 0.05). The letters a and b indicate differences between treatments (*p* < 0.05); unmarked letters indicate no significant differences (*p* > 0.05). w.w. means wet weight.

**Table 6:** Essential amino acid profile in muscle of oriental river prawn (g/100 g of fresh prawn muscle).

Amino Acid	Dietary Leucine Levels (%)					
	0.76	1.16	1.48	1.94	2.32	2.74
EAA						
Arginine	1.62 ± 0.03	1.54 ± 0.03	1.65 ± 0.02	1.56 ± 0.06	0.65 ± 0.14	1.54 ± 0.06
Histidine	0.31 ± 0.05	0.26 ± 0.02	0.32 ± 0.03	0.26 ± 0.03	0.26 ± 0.05	0.28 ± 0.05
Isoleucine	0.75 ± 0.05	0.70 ± 0.03	0.77 ± 0.01	0.63 ± 0.01	0.79 ± 0.07	0.72 ± 0.07
Leucine	1.29 ± 0.01 ^a^	1.29 ± 0.02 ^a^	1.33 ± 0.01 ^a^	1.34 ± 0.01 ^a^	1.50 ± 0.05 ^b^	1.39 ± 0.01 ^ab^
Lysine	1.51 ± 0.03	1.42 ± 0.06	1.47 ± 0.01	1.41 ± 0.03	1.52 ± 0.10	1.41 ± 0.07
Methionine	0.57 ± 0.02	0.53 ± 0.02	0.56 ± 0.01	0.51 ± 0.02	0.57 ± 0.05	0.54 ± 0.02
Phenylalanine	0.70 ± 0.03	0.66 ± 0.03	0.71 ± 0.01	0.65 ± 0.04	0.70 ± 0.08	0.69 ± 0.05
Threonine	0.55 ± 0.05	0.51 ± 0.03	0.60 ± 0.02	0.50 ± 0.05	0.58 ± 0.07	0.53 ± 0.08
Valine	0.74 ± 0.05	0.68 ± 0.03	0.75 ± 0.01	0.67 ± 0.04	0.72 ± 0.10	0.71 ± 0.06
ΣEAA	8.04 ± 0.30	7.58 ± 0.26	8.16 ± 0.09	7.52 ± 0.26	8.29 ± 0.67	7.80 ± 0.45
NEAA						
Aspartic acid	1.84 ± 0.13	1.75 ± 0.09	1.94 ± 0.02	1.66 ± 0.12	1.78 ± 0.25	1.78 ± 0.19
Serine	0.67 ± 0.01	0.64 ± 0.02	0.67 ± 0.01	0.61 ± 0.03	0.67 ± 0.06	0.65 ± 0.03
Glycine	1.51 ± 0.03	1.47 ± 0.06	1.44 ± 0.00	1.42 ± 0.04	1.53 ± 0.05	1.41 ± 0.06
Alanine	1.08 ± 0.03	1.03 ± 0.03	1.07 ± 0.01	0.95 ± 0.04	1.05 ± 0.10	1.04 ± 0.04
Cystine	0.08 ± 0.03	0.09 ± 0.00	0.17 ± 0.01	0.13 ± 0.02	0.10 ± 0.02	0.17 ± 0.03
Tyrosine	0.48 ± 0.04	0.42 ± 0.03	0.51 ± 0.02	0.45 ± 0.03	0.49 ± 0.06	0.50 ± 0.06
Glutamic acid	2.82 ± 0.11	2.68 ± 0.10	2.87 ± 0.02	2.62 ± 0.14	2.79 ± 0.32	2.73 ± 0.17
Proline	0.66 ± 0.03	0.62 ± 0.03	0.68 ± 0.02	0.57 ± 0.04	0.68 ± 0.05	0.64 ± 0.06
ΣNEAA	9.14 ± 0.26	8.69 ± 0.31	9.34 ± 0.11	8.42 ± 0.43	8.98 ± 0.85	8.90 ± 0.61
ΣEAA/ΣNEAA	0.88 ± 0.01	0.87 ± 0.01	0.87 ± 0.01	0.90 ± 0.02	0.93 ± 0.03	0.88 ± 0.01

Notes: Values are means ± SEM (n = 4). EAA, essential amino acid; NEAA, non-essential amino acid. Means in the same line with different superscripts are significantly different by Tukey’s test (*p* < 0.05). The letters a and b indicate differences between treatments (*p* < 0.05), while the unmarked corner letters indicate no significant differences (*p* > 0.05).

**Table 7:** The effect of dietary leucine levels on the muscle texture of oriental river prawn.

Index	Dietary Leucine Levels (%)					
	0.76	1.16	1.48	1.94	2.32	2.74
Hardness (g)	557.92 ± 41.45	691.16 ± 67.66	796.92 ± 169.73	440.01 ± 54.75	427.94 ± 35.5	450.63 ± 54.89
Springiness	0.30 ± 0.01	0.28 ± 0.01	0.30 ± 0.01	0.34 ± 0.02	0.33 ± 0.02	0.32 ± 0.01
Cohesiveness	0.33 ± 0.02 ^ab^	0.33 ± 0.02 ^ab^	0.37 ± 0.01 ^b^	0.29 ± 0.01 ^a^	0.32 ± 0.01 ^ab^	0.34 ± 0.01 ^ab^
Gumminess	180.12 ± 23.46	170.92 ± 8.52	150.01 ± 11.23	130.59 ± 19.32	138.28 ± 14.01	156.17 ± 20.81
Chewiness	47.19 ± 8.13	57.27 ± 3.87	54.55 ± 6.62	53.71 ± 5.40	56.9 ± 3.50	64.76 ± 8.59
Resilience	0.16 ± 0.01 ^a^	0.19 ± 0.01 ^ab^	0.17 ± 0.01 ^ab^	0.21 ± 0.02 ^b^	0.19 ± 0.00 ^ab^	0.2 ± 0.01 ^ab^
Shear Force (kg)	0.66 ± 0.07 ^b^	0.58 ± 0.07 ^ab^	0.46 ± 0.07 ^ab^	0.48 ± 0.04 ^ab^	0.36 ± 0.05 ^a^	0.35 ± 0.05 ^a^

Note: Values are means ± S.E.M (n = 4). The different corner letters in the same row indicate significant differences (*p* < 0.05), while the unmarked corner letters indicate no significant differences (*p* > 0.05).

## Data Availability

The data are available from the corresponding author on reasonable request.

## Abbreviations

The following abbreviations are used in this manuscript:

SGR	Specific growth rate
WGR	weight gain rate
FCR	Feed conversion ratio
PER	Protein efficiency ratio
PDR	Protein deposition rate
HSI	Hepatopancreas index
CF	Condition factor
TRY	Trypsin
LPS	Lipase
AMS	Amylase
MDA	Malondialdehyde
SOD	Superoxide dismutase
T-AOC	Total antioxidant capacity
LA	Lactic acid
LDH	Lactic dehydrogenase
GLU	Blood glucose

## References

[1] Cao X., Cui H., Ji X., Lu Y., Kang Q., Lu R., Zhang R., Xu X., Chen J. (2025). Branched-chain amino acids target miR-203a/fosb axis to promote skeletal muscle growth in common carp (*Cyprinus carpio*). Aquac. Nutr..

[2] Dai X., Li X., Yin D., Chen X., Wang L., Pang L., Fu Y. (2024). Identification and characterization of TOR in *Macrobrachium rosenbergii* and its role in muscle protein and lipid production. Sci. Rep..

[3] Yang H., Rahman M.M., Li X., Sharifuzzaman S.M., Leng X. (2022). Dietary leucine requirement of juvenile largemouth bass (*Micropterus salmoides*) based on growth, nutrient utilization and growth-related gene analyses. Aquaculture.

[4] Liu F., Liu Y., Tian L., Chen W., Yang H., Du Z. (2014). Quantitative dietary leucine requirement of juvenile pacific white shrimp, *Litopenaeus vannamei* (boone) reared in low-salinity water. Aquac. Nutr..

[5] Huo Y., Jin M., Sun P., Hou Y., Li Y., Qiu H., Zhou Q. (2017). Effect of dietary leucine on growth performance, hemolymph and hepatopancreas enzyme activities of swimming crab, *Portunus trituberculatus*. Aquac. Nutr..

[6] Millamena O.M., Teruel M.B., Kanazawa A., Teshima S. (1999). Quantitative dietary requirements of postlarval tiger shrimp, *Penaeus monodon*, for histidine, isoleucine, leucine, phenylalanine and tryptophan. Aquaculture.

[7] Yang C., Ye L., Liu B., Sun C., Zheng X., Miao L., Zhou Q., Jiang S. (2023). Dietary arginine requirements of oriental river prawns (*Macrobrachium nipponense*) assessed using growth and biomarkers of antioxidant capacity. Aquaculture.

[8] Worlanyo H.G., Jiang S., Yu Y., Liu B., Zhou Q., Sun C., Miao L., Lin Y., Zheng X., Saidyleigh M. (2022). Effects of dietary threonine on growth and immune response of oriental river prawn (*Macrobrachium nipponense*). Fish Shellfish Immunol..

[9] Cao S., Chen Q., Wang Q., Liu B., Zheng X., Zhou Q. (2025). Optimal tryptophan improved growth and regulated agonistic behavior of oriental river prawn, *Macrobrachium nipponense*. Aquac. Nutr..

[10] Suryawan A., Orellana R.A., Fiorotto M.L., Davis T.A. (2011). Triennial growth symposium: Leucine acts as a nutrient signal to stimulate protein synthesis in neonatal pigs. J. Anim. Sci..

[11] Wolfson R.L., Sabatini D.M. (2017). The dawn of the age of amino acid sensors for the mTORC1 pathway. Cell Metab..

[12] Zhang B., Chu L., Liu H., Xie C., Qiao S., Zeng X. (2017). Leucine supplementation in a chronically protein-restricted diet enhances muscle weight and postprandial protein synthesis of skeletal muscle by promoting the mTOR pathway in adult rats. Engineering.

[13] Song S., Kim S., Lee Y., Kim S., Lee Y., Kim W., Kim M., Jung H., Kim D., Lee K. (2026). Dietary leucine requirement and its effects on growth, immunity, antioxidant capacity and protein metabolism in pacific white shrimp, *Penaeus vannamei*. Mar. Biotechnol..

[14] Siwicki A.K., Morand M., Fuller J., Nissen S., Goryczko K., Ostaszewski P., Kazun K., Głombski E. (2003). Influence of feeding the leucine metabolite β-hydroxy-β-methylbutyrate (HMB) on the non-specific cellular and humoral defence mechanisms of rainbow trout (*Oncorhynchus mykiss*). J. Appl. Ichthyol..

[15] Zhou C., Lin H., Huang Z., Wang J., Wang Y., Yu W. (2020). Effects of dietary leucine levels on intestinal antioxidant status and immune response for juvenile golden pompano (*Trachinotus ovatus*) involved in Nrf2 and NF-κB signaling pathway. Fish Shellfish Immunol..

[16] Cheng X., Li M., Leng X., Wen H., Wu F., Yu L., Jiang M., Lu X., Gao W., Zhang W. (2021). Creatine improves the flesh quality of pacific white shrimp (*Litopenaeus vannamei*) reared in freshwater. Food Chem..

[17] Vélez E.J., Lutfi E., Azizi S., Perelló M., Salmerón C., Riera-Codina M., Ibarz A., Fernández-Borràs J., Blasco J., Capilla E. (2017). Understanding fish muscle growth regulation to optimize aquaculture production. Aquaculture.

[18] Bu Y., Wang R., Liu Y., Xing K., Zhang X., Sun Y., Zhang J. (2025). CRISPR/Cas9-mediated myostatin disruption elevates the expression of genes associated with myofiber composition and growth in *Exopalaemon carinicauda*. J. Exp. Biol..

[19] Su Q., Wei J., Wang Y., Liufu B., Li H., Mai Z., Hong K., Zhou Q., Jiao T., Mu X. (2025). Role of myostatin promoter mutations in growth and molting regulation of *Macrobrachium rosenbergii*. Front. Mar. Sci..

[20] Wang M., Guo H., Huang Y., Liu W., Wang X., Xiao K., Xiong W., Hua H., Li X., Jiang G. (2022). Dietary leucine supplementation improves muscle fiber growth and development by activating AMPK/Sirt1 pathway in blunt snout bream (*Megalobrama amblycephala*). Aquac. Nutr..

[21] Yang H., Li X., Rahman M.M., Leng X. (2023). Dietary supplementation of leucine improved the flesh quality of largemouth bass, *Micropterus salmoides* through *TOR*, *FoxO3a* and *MRFs* regulation. Aquaculture.

[22] Comesaña S., Pérez-Tierra G., Calo J., Velasco C., Conde-Sieira M., Soengas J.L. (2025). Modulation of peripheral energy metabolism through central leucine administration in rainbow trout (*Oncorhynchus mykiss*). Am. J. Physiol.-Endocrinol. Metab..

[23] Liu M., Qiao G., Wang Y., Liu S., Wang X., Yue Y., Peng S. (2024). Unveiling the molecular mechanisms regulating muscle elasticity in the large yellow croaker: Insights from transcriptomics and metabolomics. Int. J. Mol. Sci..

[24] AOAC (2005). Official Methods of Analysis of AOAC International.

[25] Muhammad A.M., Yang C., Liu B., Sun C.X., Miao L.H., Zheng X.C., Pan L.K., Xia D., Zhou Q.L. (2025). Comparative analysis of meat quality and hindgut microbiota of cultured and wild bighead carp (*Hypophthalmichthys nobilis*, Richardson 1845) from the Yangtze River area. Microogganisms.

[26] He C.Q., Geng H.Y., Qin Y.W., Yang P., Wang W.Q., Mai K.S., Song F. (2023). Dietary xanthophyll improved growth, antioxidant, pigmentation and meat quality in the southern catfish (*Silurus soldatovi meridionalis* Chen). Anim. Nutr..

[27] Yang H., Xu Z., Xu X., Rahman M.M., Li X., Leng X. (2022). Transcriptomic and biochemical analyses revealed the improved growth, lipid metabolism, and flesh quality of grass carp (*Ctenopharyngodon idellus*) by dietary *eucommia ulmoides* bark and leaf supplementation. J. Anim. Sci..

[28] Livak K.J., Schmittgen T.D. (2001). Analysis of relative gene expression data using real-time quantitative PCR and the 2^−ΔΔCT^ method. Methods.

[29] Zhou Q., Jiang S., Xiong Y., Liu B., Sun C., Jiang Z., Fu H. (2020). Fishmeal level affects growth performance of *Macrobrachium nipponense* via regulating protein and lipid metabolism. Aquac. Int..

[30] Ding Z., Luo N., Kong Y., Li J., Zhang Y., Cao F., Ye J. (2016). Scavenger receptor class B, type I, a CD36 related protein in *Macrobrachium nipponense*: Characterization, RNA interference, and expression analysis with different dietary lipid sources. Int. J. Genom..

[31] Hu Y., Fu Y., Jin S., Fu H., Qiao H., Zhang W., Jiang S., Gong Y., Xiong Y., Wu Y. (2021). Comparative transcriptome analysis of lethality in response to RNA interference of the oriental river prawn (*Macrobrachium nipponense*). Comp. Biochem. Physiol. Part D Genom. Proteom..

[32] Wei Z., Zhuang Y., Liu X., Zou D., Mai K., Sun Z., Ye C. (2023). Leucine promotes protein synthesis of juvenile white shrimp *Litopenaeus vannamei* through TOR signaling pathway. Aquaculture.

[33] Song M., Yu Q., Li E., Song Y., Cai X., Huang Y., Qin C., Wang X., Qin J., Chen L. (2024). Leucine improves dietary protein use efficiency by regulating protein synthesis by activating amino acid transporters and the mTORC1 pathways in Chinese mitten crab (*Eriocheir sinensis*). Aquaculture.

[34] Fulton T.L., Johnstone J.N., Tan J.J., Balagopal K., Dedman A., Chan A.Y., Johnson T.K., Mirth C.K., Piper M.D.W. (2024). Transiently restricting individual amino acids protects *Drosophila melanogaster* against multiple stressors. Open Biol..

[35] Xia Y., Ye J., Zhou Z., Zhang Y., Wu C., Ming J. (2014). Study on the optimal levels of dietary leucine and isoleucine for juvenile Chinese mitten crabs, *Eriocheir sinensis*. Acta Hydrobiol. Sin..

[36] Niu X., Qian X., Feng H., Yi K., Li D., Chen W., Ye J. (2021). Growth and metabolic responses of grouper juveniles (*Epinephelus coioides*) fed diets containing varying levels of leucine. Aquaculture.

[37] Tan X., Lin H., Huang Z., Zhou C., Wang A., Qi C., Zhao S. (2016). Effects of dietary leucine on growth performance, feed utilization, non-specific immune responses and gut morphology of juvenile golden pompano *Trachinotus ovatus*. Aquaculture.

[38] Wang S., Liu S., Zhang S., Lu S., Han S., Jiang H., Liu H., Wang C. (2026). Dietary leucine supplementation enhances growth performance, digestive enzyme activity, and intestinal barrier function in triploid rainbow trout (*Oncorhynchus mykiss*) fed plant-protein based diets. Aquac. Rep..

[39] Zhu J., Jiang G., Xu W., Li X., Zhang W., Liu W., Yang W. (2014). Optimal dietary methionine Level of *Macrobrachium nipponense*. J. Econ. Anim..

[40] Deng Y., Jiang W., Liu Y., Jiang J., Kuang S., Tang L., Tang W., Wu P., Zhang Y., Zhou X. (2014). Differential growth performance, intestinal antioxidant status and relative expression of Nrf2 and its target genes in young grass carp (*Ctenopharyngodon idella*) fed with graded levels of leucine. Aquaculture.

[41] Ahmad I., Ahmed I., Dar N.A. (2021). Effects of dietary leucine levels on growth performance, hematobiochemical parameters, liver profile, intestinal enzyme activities and target of rapamycin signalling pathway related gene expression in rainbow trout, *Oncorhynchus mykiss* fingerlings. Aquac. Nutr..

[42] Jiang W., Deng Y., Zhou X., Liu Y., Jiang J., Kuang S., Tang L., Tang W., Wu P., Zhang Y. (2017). Towards the modulation of oxidative damage, apoptosis and tight junction protein in response to dietary leucine deficiency: A likely cause of ROS-induced gill structural integrity impairment. Fish Shellfish Immunol..

[43] Ren M., Habte-Tsion H.-M., Liu B., Miao L., Ge X., Xie J., Liang H., Zhou Q., Pan L. (2015). Dietary leucine level affects growth performance, whole body composition, plasma parameters and relative expression of TOR and TNF-ɑ in juvenile blunt snout bream, *Megalobrama amblycephala*. Aquaculture.

[44] Zhang Y., Liu Y., Tang X., Zhu X., Qian Q., Gao X., Jiang Q., Zhang X. (2025). Insights into the roles of vitamin D3 on the antioxidant capacity, flesh quality, and myofiber development of giant freshwater prawns (*Macrobrachium rosenbergii*). Aquac. Rep..

[45] Miao L., Zhang Y., Lin Y., Liu B., Ge X. (2021). Appropriate leucine supplementation promotes glucose metabolism and enhances energy homeostasis in juvenile crucian carp (*Carassius auratus gibelio* var. CAS III). Comp. Biochem. Physiol. Part D Genom. Proteom..

[46] Yun Y., Song D., He Z., Mi J., Wang L., Nie G. (2022). Effects of methionine supplementation in plant protein based diet on growth performance and fillet quality of juveniles yellow river carp (*Cyprinus carpio haematopterus*). Aquaculture.

[47] Deng Y., Jiang W., Liu Y., Qu B., Jiang J., Kuang S., Tang L., Tang W., Wu P., Zhang Y. (2016). Dietary leucine improves flesh quality and alters mRNA expressions of Nrf2-mediated antioxidant enzymes in the muscle of grass carp (*Ctenopharyngodon idella*). Aquaculture.

[48] Han Y., Han R., Koshio S., Ishikawa M., Yokoyama S., Gao J. (2014). Interactive effects of dietary valine and leucine on two sizes of Japanese flounder *Paralichthys olivaceus*. Aquaculture.

[49] Yamamoto T., Shima T., Furuita H. (2004). Antagonistic effects of branched-chain amino acids induced by excess protein-bound leucine in diets for rainbow trout (*Oncorhynchus mykiss*). Aquaculture.

